# Periosteal Skeletal Stem Cells and Their Response to Bone Injury

**DOI:** 10.3389/fcell.2022.812094

**Published:** 2022-03-24

**Authors:** Nian Zhang, Liru Hu, Zhiwei Cao, Xian Liu, Jian Pan

**Affiliations:** State Key Laboratory of Oral Diseases, Department of Oral and Maxillofacial Surgery, National Clinical Research Center for Oral Diseases, West China Hospital of Stomatology, Sichuan University, Chengdu, China

**Keywords:** skeletal stem cell, periosteum, fate decision, lineage tracing, bone repair

## Abstract

Bone exhibits remarkable self-repair ability without fibrous scars. It is believed that the robust regenerative capacity comes from tissue-resident stem cells, such as skeletal stem cells (SSCs). Roughly, SSC has two niches: bone marrow (BM) and periosteum. BM-SSCs have been extensively studied for years. In contrast, our knowledge about periosteal SSCs (P-SSCs) is quite limited. There is abundant clinical evidence for the presence of stem cell populations within the periosteum. Researchers have even successfully cultured “stem-like” cells from the periosteum *in vitro*. However, due to the lack of effective markers, it is difficult to evaluate the stemness of real P-SSCs *in vivo*. Recently, several research teams have developed strategies for the successful identification of P-SSCs. For the first time, we can assess the stemness of P-SSCs from visual evidence. BM-SSCs and P-SSCs not only have much in common but also share distinct properties. Here, we provide an updated review of P-SSCs and their particular responses to bone injury.

## Introduction

The periosteum, a specialized membranous structure covering the surface of cortical bones, is a crucial component of diaphysis growth during intramembranous and endochondral bone development (Olsen et al., 2000). Bone tissue is constantly remodeling throughout the lifetime. In addition to the essential function of nourishing bones, the periosteum also maintains bone homeostasis by forming bones directly underneath it (Ferretti and Mattioli-Belmonte, 2014). Although the bones are formed by either intramembranous ossification or endochondral ossification, there appears to be a difference in periosteal structure between the two. Histologically, the periosteum consists of two layers serving as the attachment site of skeletal muscles (Allen et al., 2004). The outer layer (fibrous layer) comprises collagen, elastin, and scarce cells (Buckwalter and Cooper, 1987; Squier et al., 1990; Dwek, 2010). In addition, it also contains a high density of distinct microvascular networks called the “umbilical cord of bone” (Chanavaz, 1995) and linear neuron fibers (Mach et al., 2002; Matsuo et al., 2019). The inner layer (cellular layer) contains a slew of fibroblast-like cells. It is a highly vascularized structure with a high density of microvessels (Dwek, 2010), also known as the cambium layer, which is thicker in early life and then becomes thinner with age (Uddströmer, 1978).

Numerous studies from clinical trials and animal models have shown the osteogenic capacity of the adult periosteum. Periosteal grafts have been successfully used to reconstruct large quantities of bone tissue with high quality in the treatment of pseudarthrosis or infected sites (Masquelet et al., 1988), large bone defects (Lapierre et al., 1991; Gallardo-Calero et al., 2019), fracture non-union (Jaloux et al., 2020), and osteoradionecrosis (Yachouh et al., 2010). The periosteal reaction is another example. The periosteum may be elevated from the cortex in response to various insults, such as trauma, infection, and tumors. As a result, the periosteum can form new laminated or onion skin-like bones (Rana et al., 2009), which are also critical for mechanical loading. Femurs covered with periosteum showed significantly higher bone strength than the periosteum-stripped ones (Yiannakopoulos et al., 2008). The ablation of periosteal skeletal stem cells (P-SSCs) severely interfered with routine maintenance of bone homeostasis and mechanical loading-induced bone formation (Moore et al., 2018).

In bone repair, the regenerated cells may come from two resources, one from the circulation and the other from local tissues. Whether circulating “stem cells” are involved in the production of osteoblasts has not been proven until recently. Ransom et al. (2018) surgically connected the blood vessels from green fluorescent protein (GFP)-labeled and non-GFP-labeled littermate mice so that they shared a common circulation system. Then, the non-GFP mice were treated with distraction osteogenesis (DO) surgery. Twenty-nine days after surgery, no GFP + cells were detected in the distraction callus (Ransom et al., 2018). In another study, fractured femurs from a genetically labeled donor were transplanted into the renal capsule of a wild-type host. After 14 days, the regenerated cells were confirmed as the donor origin (Duchamp de Lageneste et al., 2018). These studies provided convincing evidence ruling out the contribution from circulation.

After the exclusion of circulation contribution, the regenerated cells were most likely of local origin. The next step is to determine whether they come from the periosteum, cortical bone, or bone marrow (BM). To solve this question, Colnot (2009) designed a series of bone graft transplantation experiments. Bone grafts from RosaLac-Z mice with or without periosteum were transplanted into wild-type mice. By tracing the Lac-Z-positive cells, it was concluded that repairing cells came from adjacent tissues; cells derived from the periosteum were always found on the periosteal surface; cells derived from the BM were always found within the marrow cavity; and cortical bone involvement was very limited. In the callus, cartilage cells are almost always derived from the periosteum, while osteoblasts are derived from both the periosteum and BM (Colnot, 2009; Wang et al., 2019). In a similar study, the authors stated that approximately 70% of the total regenerated cells came from the periosteum (Zhang et al., 2005). Although both the bone marrow and periosteum contribute to the formation of new bone, the quantity is different. Because the majority of bone callus tissue is located beneath the periosteum, it is no surprise to find that most of the regeneration cells come from the periosteum. As shown by Kojimoto et al. (1988), bone regeneration is highly dependent on the periosteum rather than on the bone marrow. When the periosteum was removed during DO, bone regeneration was markedly disturbed. However, in contrast, scraping of BM had no pronounced effect (Kojimoto et al., 1988). Although in cases when the periosteum were removed, the cells from adjacent skeletal muscle tissue could be involved in bone repair as a compensation (Julien et al., 2021; Liu et al., 2011). When the periosteum is in its position, its regeneration capacity is sufficient, and other alternative cellular sources are not required (Liu et al., 2011).

All of these studies demonstrated the strong regenerative capacity of the periosteum. Furthermore, the capacity is believed to come from P-SSCs. In this review, we summarized the current knowledge of periosteal SSCs for their origin, identity, properties, and special response to injuries.

## The Concept of Skeletal Stem Cells

SSCs are a concept developed from mesenchymal stem cells (MSCs). The term MSCs was first introduced by Caplan (1991) to describe a population of cells in the bone marrow that possesses trilineage (adipogenic, osteogenic, and chondrogenic) differentiation capacity (Caplan, 1991; Dominici et al., 2006). MSCs have long been considered to be a pure cell population, and each cell has trilineage differentiation potential. However, MSCs actually have high heterogeneity, and every individual cell is not equal in its differentiation capacity (Viswanathan et al., 2019). Leptin receptor (LepR) is a widely accepted marker of adult MSCs. In their physiological state, BM-LepR + cells give rise to bone and adipose tissue (Zhou et al., 2014). In a recent study, LepR + cells were divided into two subgroups: one enriched for osteogenic genes and the other enriched for adipogenic genes at the transcription level (Tikhonova et al., 2019). Under adipogenic induction, only already transcribed adipogenic genes would respond; others remained relatively quiescent (Tikhonova et al., 2019). Both Gremlin1 (Worthley et al., 2015) and PTHrP (Mizuhashi et al., 2018) have labeled a subgroup of MSCs residing within the growth plates. These cells could be differentiated into chondrogenic, osteogenic, and adipogenic lineage cells *in vitro*. However, they only generated bone, cartilage, reticular stromal, and no adipose cells in lineage tracing experiments (Worthley et al., 2015) (Mizuhashi et al., 2018). The same results were obtained from ectopic transplantation experiments. Chan et al. (2015, 2018) isolated CD45−Ter-119−Tie2−AlphaV + Thy−6C3−CD105−CD200+ cells in mice (Chan et al., 2015) and PDPN + CD146-CD73 + CD164+ cells in human growth plates (Chan et al., 2018) and transplanted them into the renal capsule. Only bone, cartilage, and stroma but not adipose tissue were generated. These data support the existence of at least two subgroups of MSCs: one poised for adipogenic differentiation and the other poised for osteogenic differentiation. Although MSCs can undergo trilineage differentiation *in vitro*, this phenomenon is induced by exogenous stimuli. In the absence of exogenous stimulation, the situation is different *in vivo*. In fact, large areas of the chromatin landscape need to be reshaped as osteogenic MSCs are driven to adipogenic differentiation (Meyer et al., 2016; Rauch et al., 2019). Therefore, each MSC is likely to carry a genetic preprogram that suggests a restricted differentiation direction. In some situations, such as in a living organism without exogenous stimuli, they are more likely to behave in a preprogrammed manner. The seemingly trilineage differentiation potential of MSCs is more likely to aggregate potential compounds of distinct cell types.

By focusing on the differentiation potential, “SSCs” are proposed to define a group of cells that already carry an osteogenic program that could differentiate into skeletal lineage (bone and cartilage) cells, excluding adipogenic lineages. However, the concept of “SSCs” is still in the development stage; “bone marrow “MSCs” and “SSCs” are currently used interchangeably. Only recently has the concept of SSCs been extrapolated from BM/growth plate (GP) to the periosteum.

## Developmental Origin of the Periosteal-Skeletal Stem Cells

**FIGURE 1:**
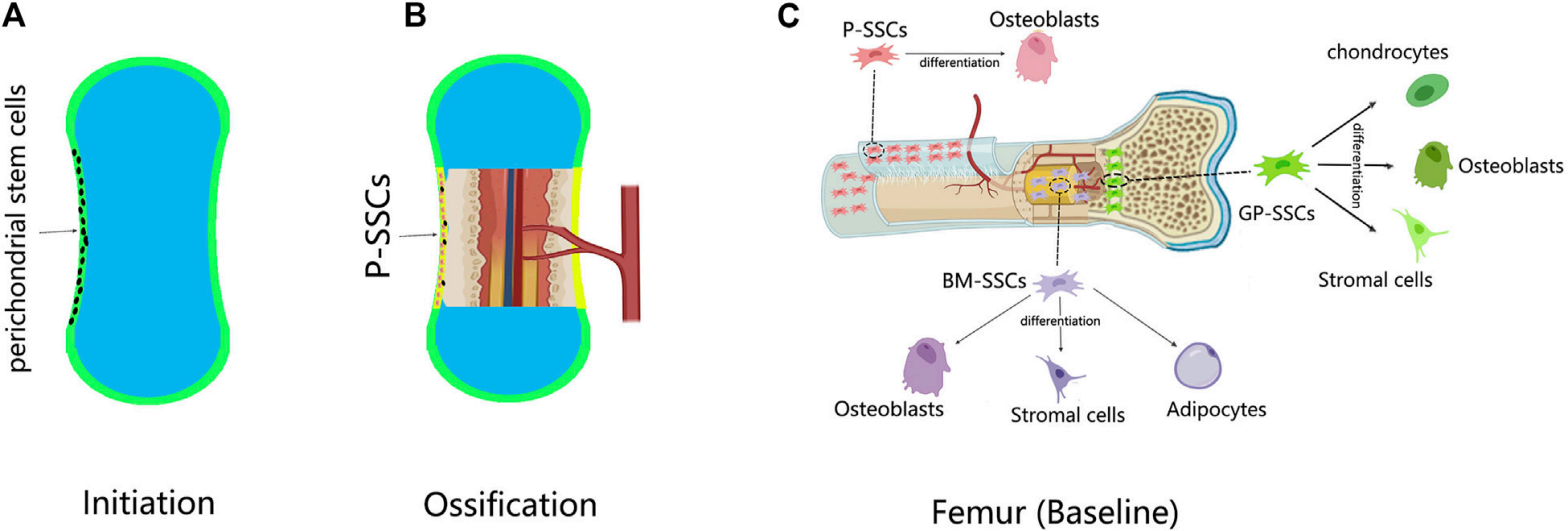
The development of P-SSCs and their differentiation capacity. **(A)** In the limb development, a cartilage template is first established; the perichondrium contains undifferentiated mesenchymal cells. **(B)** Ossification begins when the blood vessel invades the center of the cartilage; the perichondrium is changed into periosteum. The perichondral mesenchymal cells give rise to P-SSCs. **(C)** Skeletal stem cells (SSCs) in different sites of the femur have distinct differentiation abilities. Compared with bone marrow-SSCs (BM-SSCs) and growth plate-SSCs (GP-SSCs) the periosteal SSCs (P-SSCs) can only give rise to osteoblasts at baseline.

Skeletons are formed by either intramembranous ossification or endochondral ossification. In intramembranous ossification, mesenchymal cells condense at the site of the future periosteum. Transcription factor 2 (Runx2) and osteogenic factor (Sp7) are then expressed in sequence, causing mesenchymal cells to differentiate directly into osteoblasts (Long, 2011). Undifferentiated mesenchymal cells form the periosteum, and some of them form P-SSCs (Ochareon and Herring, 2011) (Figures 1A,B).

In endochondral ossification, mesenchymal cells condense at the position of future bones (Akiyama et al., 2005). By expressing SRY-box 9 (Sox9), mesenchymal cells differentiate into chondrocytes to form cartilage templates (Akiyama et al., 2002; Akiyama et al., 2005; Long and Ornitz, 2013). The undifferentiated mesenchymal cells at the periphery form the perichondrium. At the same time, blood vessels invade the center of the cartilage template, forming the bone marrow cavity and transitioning from cartilage to bone tissue (Long and Ornitz, 2013). As ossification progresses, the perichondrium is reshaped into the periosteum (Kronenberg, 2007). During this time, the perichondral stem cells become P-SSCs. Therefore, whether P-SSCs are derived from local skeletal elements or from blood has not been elucidated until recently. Duchamp de Lageneste et al. (2018) transplanted unvascularized femur cartilage templates from Prx1-Cre and YFPfl/+ donors to the renal capsule of wild-type hosts. After 8 weeks, the cartilage templates developed into bone tissue, and the P-SSCs came from the donor (Duchamp de Lageneste et al., 2018). Lineage tracing experiments also provided evidence from another aspect. Hox11 is regionally expressed in the perichondrium of zeugopod limbs during the embryonic stage. Hox11-expressing cells persist in the periosteum continuously, giving rise to SSCs and osteoprogenitors from the embryonic to adult stage (Pineault et al., 2019). These experiments demonstrated the local origin of P-SSCs.

## Finding a Good Marker for Periosteal-Skeletal Stem Cells

P-SSCs and BM-SSCs are comparable to a great degree. Some markers, such as Nestin and LepR, used to identify BM-SSCs also label P-SSCs (Gao et al., 2019). BM, however, appears to be a much larger pool of cells (Méndez-Ferrer et al., 2010; Zhou et al., 2014). In one study, distinguishing between P-SSCs and BM-SSCs using these markers was difficult. A strategy of combined markers (CD45−Ter-119−Tie2−AlphaV + Thy−6C3−CD105−CD200+) provided by Chan et al. (2015) to identify GP-SSCs was also effective for P-SSCs (Chan et al., 2015; Ransom et al., 2018; Tournaire et al., 2020). However, the population in the periosteum contains approximately 7%–8% mature osteoblasts (Matthews et al., 2021). α-SMA is another well-studied marker that was originally a pericyte marker. Pericytes exhibit “stemness” in various tissues, and they are considered tissue-resident stem cells (Crisan et al., 2008; Wong et al., 2015). α-SMA has been proven to label some (not all) SSCs in the periosteum (Diaz-Flores et al., 1992; Matthews et al., 2014). However, a recent study uncovered the heterogeneity of periosteal α-SMA+ cells by single-cell RNA-seq (Matthews et al., 2021). The periosteal α-SMA+ cell population comprises three clusters: P-SSCs, fibroblasts, and perivascular cells (Matthews et al., 2021). Therefore, α-SMA alone is not appropriate for serving as a P-SSC marker. MX1 is a BM-SSC marker with some limitations. Mx1-Cre is broadly expressed in other lineages but is not expressed under normal circumstances (Park et al., 2012). Ortinau et al. (2019) combined these two strategies and successfully identified MX1 + α-SMA+ cells as P-SSCs.

Specifically, this combination only labeled P-SSCs, and no double-positive cells were seen in the BM (Ortinau et al., 2019). Prrx1 is a broad skeletal mesenchymal cell marker (ten Berge et al., 1998; Peterson et al., 2005) that is expressed in both the endosteum and periosteum in the early postnatal period. With increasing age, Prrx1 expression is gradually restricted in the periosteum to mark P-SSCs (Esposito et al., 2020). Another marker is the cysteine protease cathepsin K (CTSK). Historically, CTSK has been used as a marker of osteoclasts; however, this application is site specific. In the BM, CTSK-lineage cells were tartrate-resistant acid phosphatase (TRAP) positive, indicating osteoclasts; however, the CTSK-lineage cells in the periosteum were TRAP negative. The periosteal CTSK-lineage cells include three clusters: P-SSCs (CD200+ CD105−), periosteal progenitor 1 (PP1) (CD200−CD105−), and periosteal progenitor 2 (PP2) (CD105 + CD200_variable_). Among them, P-SSCs are the most stem-like cells, and they are the precursors of PP1 and PP2 (Debnath et al., 2018). Ideally, the markers should be able to separate P-SSCs from BM-SSCs, thus making α-SMA + MX1, Prrx1, and CTSK good candidates.

Characterization of P-SSCs by gene expression analysis revealed that the α-SMA + MX1+ P-SSCs could highly express CD105, CD140a, Cxcl12, LepR, and Grem1 (Ortinau et al., 2019). Prrx1-lineage P-SSCs overexpressed PDGFRα, Grem1, Cxcl12, Nestin, and NG2 but not LepR (Duchamp de Lageneste et al., 2018). The CTSK-lineage P-SSC population contains Gremlin1+ and Nestin+ subsets but not LepR, CD140a, or CD146 (Debnath et al., 2018).

In conclusion, the markers that identify BM-SSCs may also identify P-SSCs. To date, α-SMA + MX1, Prrx1, and CTSK are P-SSC-specific markers, and there are overlapping subsets among them.

## The Stemness of Periosteal-Skeletal Stem Cells

“Stemness” means the ability to proliferate, self-renew, and differentiate. Traditionally, the colony-forming ability on plastic dishes is referred to as “proliferative/self-renewal,” and the multilineage differentiation ability in certain induction media is called “multipotency.” However, some authors have noted the shortcomings of these definitions. Colony-forming assays or expansion cultures demonstrate not self-renewal but rather proliferation capacity (Bianco and Robey, 2015). *In vitro* differentiation assays proved the plasticity rather than the differentiation capacity. To eliminate the effect of exogenous factors, Bianco and Robey (2015) suggested that the differentiation capacity should be assessed by heterotopic transplantation of non-doctored and non-induced cultures. In this review, we evaluated the stemness of SSCs mainly by their behaviors *in vivo.*

The self-renewal capacity of Mx1+αSMA + P-SSCs was evaluated by serial transplantation experiments. The donor Mx1+αSMA + P-SSCs were sorted and transplanted into calvarial injury sites of hosts with Matrigel. Four weeks later, the Mx1 + αSMA + P-SSCs repopulated and generated new bone. Following the second transplantation, the retransplanted Mx1 + αSMA + P-SSCs also maintained repopulation and differentiation capacity (Ortinau et al., 2019). Similarly, the self-renewal capacity of CTSK-lineage P-SSCs was analyzed by two successive rounds of heterotopic transplantation. Donor CTSK-lineage P-SSCs were transplanted into the mammary fat pad and kidney capsule of female hosts. In both rounds, the CTSK-lineage P-SSCs self-renewed and rebuilt the P-SSC pool. The CTSK-lineage P-SSCs maintained their immunophenotype and differentiation capacity after the last transplantation (Debnath et al., 2018).

For the differentiation capacity, the same conclusion was drawn from different SSC lineage experiments. Nestin-lineage (Tournaire et al., 2020), CTSK-lineage (Debnath et al., 2018), and Mx1 + αSMA + (Ortinau et al., 2019) P-SSCs only generated bone without cartilage or stroma in transplantation. This observation coincided with the intramembranous ossification function mediated by the periosteum (Figure 1C).

In conclusion, P-SSCs are true tissue-resident stem cells with the capacity to proliferate, self-renew, and differentiate into osteolineage cells.

## Periosteal-Skeletal Stem Cells vs. Bone Marrow-Skeletal Stem Cells

Considering how closely the BM is related to the periosteum, it is interesting to compare them. *In vitro*, P-SSCs grew faster, and secondary colony formation efficiency was higher at the same density of P-SSCs and BM-SSCs (Duchamp de Lageneste et al., 2018). In transplantation assays, BM-SSCs displayed a typical endochondral ossification model generating bone, cartilage tissue, and hematopoietic stroma. P-SSCs represent an intramembranous ossification model, producing only bone tissue without cartilage and hematopoietic stroma (Debnath et al., 2018) with a higher migration capacity. In wound healing assays, the wound closure time of P-SSCs was only approximately half that of BM-SSCs (Debnath et al., 2018). *In vivo*, intravital imaging showed that the Mx1 + αSMA + P-SSCs moved toward the injury site immediately after wounding, whereas MX1 + BM-SSCs remained *in situ* (Ortinau et al., 2019). When P-SSCs and BM-SSCs were transplanted into the bone fracture site, the P-SSCs quickly penetrated into the center of the callus, while the BM-SSCs stayed at the periphery of the callus (Duchamp de Lageneste et al., 2018). At the gene expression level, P-SSCs were enriched in genes related to “stemness,” “limb development,” and “extracellular matrix (ECM),” while BM-SSCs were enriched in “downregulation of stemness,” “bone resorption,” “immune cells,” and “hematopoietic stem cells” (Duchamp de Lageneste et al., 2018). The gene expression patterns coincide with their distinct microenvironment. The BM is an immunological and hematopoietic organ; preserving immunological homeostasis and supporting hemopoiesis are also major functions of BM-SSCs (Méndez-Ferrer et al., 2010; Isern et al., 2014). This is why genes associated with hematopoiesis and immune response are enriched in BM-SSCs but not in P-SSCs. However, when we focus specifically on “stemness,” P-SSCs appear to be the better choice.

In conclusion, compared with BM-SSCs, P-SSCs have higher growth and migration potential, but their differentiation capacity is more restricted in osteolineages.

## The Contribution of Periosteal-Skeletal Stem Cells to Bone Repair

As previously mentioned, P-SSCs are considered to be a major contributor to bone regeneration. In Prrx1-Cre, after activated recombination of mTmG mice 3 days after fracture (Esposito et al., 2020), all newly regenerated cells in the callus were derived from Prrx1+ cells (Duchamp de Lageneste et al., 2018).

Conditional ablation of Prrx1+ cells in Prx1-Cre^ER^ DTA mice led to deficient healing with much less bone and cartilage content (Esposito et al., 2020). Similarly, MX1 + αSMA + P-SSCs contributed to approximately 20% of chondrocytes and 80% of new osteoblasts in the fracture callus (Ortinau et al., 2019). Conditional ablation of periosteal MX1+ cells led to significantly delayed healing and osteoblast reduction (Ortinau et al., 2019). Ablation of αSMA + cells either at the beginning or throughout the first 8 days in fracture healing led to reduced callus size formation and delayed mineralization (Matthews et al., 2021). By conditionally deleting OSX, a key osteogenic factor in the CTSK-Cre (Nakashima et al., 2002) CTSK + P-SSC osteogenic differentiation pathway was blocked. Osx^fl/fl^ mice exhibited hypomineralization of the skull, uneven periosteal surfaces, and extensive linear intracortical pores (Debnath et al., 2018). Under injury conditions, these mice showed a markedly high risk of fracture non-union with defects in mineralization and an increased volume of cartilage in the callus (Debnath et al., 2018).

In conclusion, P-SSCs are the major sources of repairing cells in bone repair. The osteogenic differentiation capacity of these cells is critical to successful bone healing.

## The Special Response of Periosteal-Skeletal Stem Cells to Different Bone Regeneration Models

In bone regeneration, endochondral and intramembranous ossification patterns occur simultaneously. Endochondral ossification predominated in unstable fracture models, but some direct bone formation and fibrochondrogenesis were also involved. Intramembranous ossification predominated in stable fracture, bone defect, and DO models (Runyan and Gabrick, 2017). In unstable fracture healing, cartilage scaffolds are first constructed and then gradually replaced with bone (Ferguson et al., 1999). Almost no cartilage is seen in intramembrane bone regeneration models (Jazrawi et al., 1998; Choi et al., 2002). P-SSCs respond differently to these patterns, but approximately 20% of available chromatin site alterations are common in fractures and DO models, including clusters related to general stress and inflammatory responses, such as HIF, VEGF, and IL signaling (Ransom et al., 2018). Here, we mainly focused on the modeled special responses of P-SSCs.

As we have discussed before, the periosteum not only mediates intramembranous ossification in physiological conditions but is also chondrogenic in fractures; this is a longstanding contradiction. In ectopic transplantation, Prrx1-, MX1 + αSMA-, and CTSK-labeled P-SSCs not only produced cartilage-less bone but also contributed approximately 100%, 20%, and 50% of the total chondroblasts in the fracture callus, respectively (Debnath et al., 2018; Duchamp de Lageneste et al., 2018; Ortinau et al., 2019).

Interestingly, the CTSK-lineage P-SSCs isolated from normal periosteum were osteoblastic only, but the P-SSCs isolated from the fractured periosteum were both osteoblastic and cartilaginous, and they could give rise to bone and cartilage in capsule transplantation (Debnath et al., 2018), indicating that a transition occurs in the P-SSCs. Genetically, P-SSCs upregulate cartilage formation-associated genes in fractures, such as Sox9 (Ransom et al., 2018), making the gene expression profiles similar to those of BM-SSCs. The fate shifting of P-SSCs in fractures has been observed, but its regulatory mechanism remains unclear. Some evidence points to the HIF and BMP pathways, but further investigation is required (Nakahara et al., 1990; Hanada et al., 2001; Ueno et al., 2001; Tsuji et al., 2006; Eyckmans et al., 2010; Chan et al., 2015).

**FIGURE 2:**
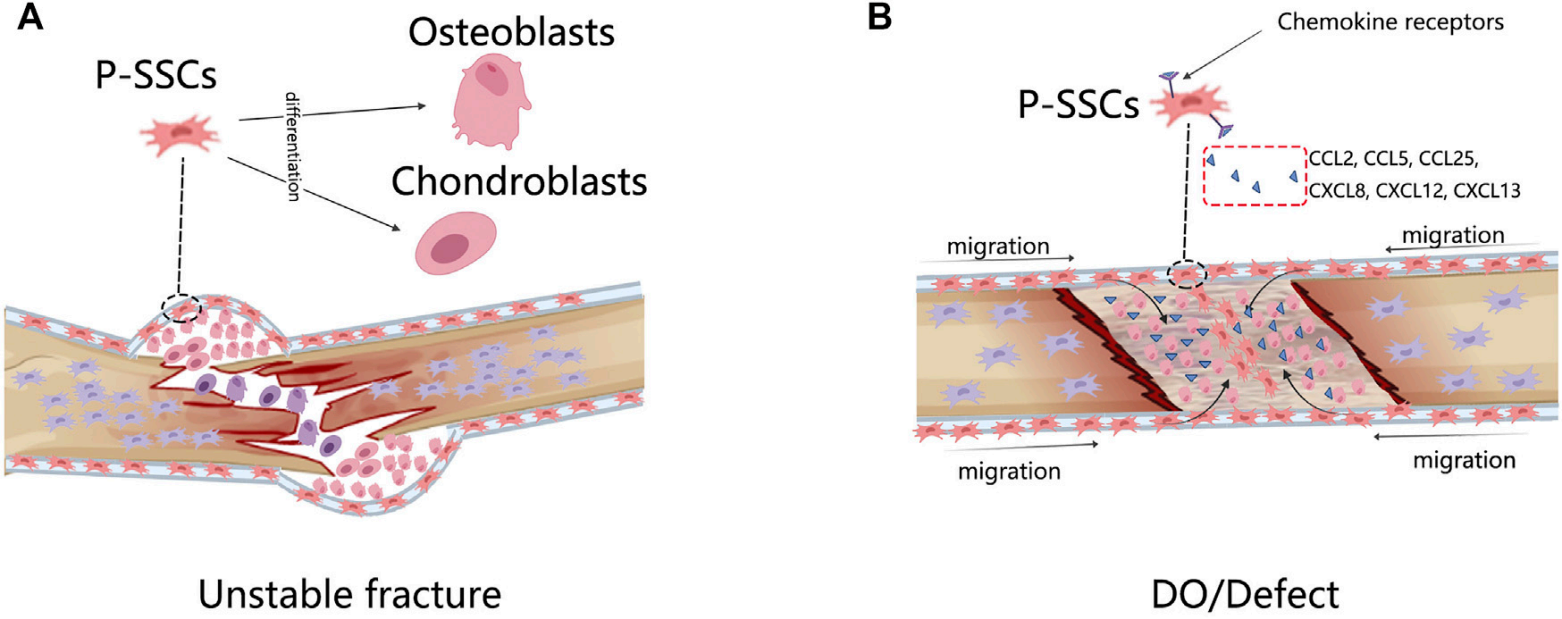
The response of P-SSCs in different bone injury models. **(A)** In unstable fracture condition, P-SSCs acquire the capacity to differentiate into chondrocytes. **(B)** In DO or defected condition, P-SSCs showed high migratory capabilities. They are recruited into the bone defect site in response to special migration signals.

The most significant difference between the fracture model and the DO model is that the DO model produces a larger bone tissue volume. The rate of bone growth during DO is equivalent to that of the fetus and is four–eight times that of adolescents (Steinbrech et al., 2000; Choi et al., 2002). Unlike the unstable fracture model that demonstrates the plasticity of P-SSCs, the DO model displays other sides of P-SSCs. Runx2 and Sox9 are key triggers of osteogenesis and chondroblast differentiation, with high chromatin accessible in fracture P-SSCs but not in DO (Ransom et al., 2018), indicating that P-SSCs are not in a poised differentiation state in DO but are ready to differentiate in fractures. The genes specifically enriched in P-SSCs during DO are associated with vascularization, adhesion, migration, and responses to mechanical stimulation (Ransom et al., 2018). Disruption of the mechanotransduction pathway FAK led to non-oriented migration of the P-SSCs and failure of DO (Ransom et al., 2018). These data indicated that the primary response of P-SSCs in DO had a close relationship with their migration within the periosteum. Human P-SSCs have been reported to express receptors from chemokine subfamilies CC, CXC, CX3C, and C, which respond to CCL2, CCL25, CXCL8, CXCL12, and CXCL13 (Stich et al., 2008). In mice, MX1 + αSMA + P-SSCs induced high expression of CCR5 and recruitment to the bone defect site (Ortinau et al., 2019). Blocking the CCL5–CCR5 interaction severely interrupts the bone healing process (Ortinau et al., 2019) (Figure 2).

In conclusion, during unstable fracture healing, osteogenic unipotent P-SSCs become osteogenic and chondrogenic bipotent. The complete the migration capability of the P-SSCs is fundamental to successful DO.

## Discussion

In this review, the research progress on P-SSC properties in recent years was summarized. P-SSCs are a population of cells with high proliferation, migration, and osteogenic potential that exhibit plasticity in fractures, but the mechanism regulating this cell fate transition has not yet been fully clarified. The high mobility of P-SSCs is critical to DO. Thoroughly understanding the properties of P-SSCs and their behavior in injury is the cornerstone for their potential clinical application.

Since the discovery of tissue-resident stem cells, they have been widely used in both fundamental and clinical research. BM-SSCs have shown plasticity *in vitro*, and subsequently, many research groups have adopted BM-SSC therapy with the hope that BM-SSCs can cure diseases by differentiating into local cell types. However, BM-SSCs rarely or never differentiate into cell types outside the skeletal lineage (Caplan, 2017). Within the skeletal lineage, when considering reconstruction of the cartilage, synovium-derived stem cells showed a higher chondrogenic potential than BM-SSCs (Sasaki et al., 2018). Cartilage regeneration using BM-SSCs requires genetic modification or induction (Chan et al., 2015), but perichondrium-derived stem cells could directly form cartilage (Kobayashi et al., 2011). Similarly, P-SSCs appear to be a better choice than BM-SSCs for bone tissue reconstruction, where we do not want to see too much cartilage. Tissue-resident stem cells carry tissue-specific memories. Therefore, we need to be very careful when trying to use SSCs as a therapy. Before transplantation, we need to determine whether cartilage or bone is needed. In other words, the best cells should be chosen for the best treatment.

## References

[B1] Akiyama H. Chaboissier M.-C. Martin J. F. Schedl A. de Crombrugghe B. (2002). The Transcription Factor Sox9 Has Essential Roles in Successive Steps of the Chondrocyte Differentiation Pathway and Is Required for Expression of Sox5 and Sox6. Genes Dev. 16 (21), 2813–2828. 10.1101/gad.1017802 12414734 PMC187468

[B2] Akiyama H. Kim J.-E. Nakashima K. Balmes G. Iwai N. Deng J. M. (2005). Osteo-chondroprogenitor Cells Are Derived from Sox9 Expressing Precursors. Proc. Natl. Acad. Sci. 102 (41), 14665–14670. 10.1073/pnas.0504750102 16203988 PMC1239942

[B3] Allen M. R. Hock J. M. Burr D. B. (2004). Periosteum: Biology, Regulation, and Response to Osteoporosis Therapies. Bone 35 (5), 1003–1012. 10.1016/j.bone.2004.07.014 15542024

[B4] Bianco P. Robey P. G. (2015). Skeletal Stem Cells. Development 142 (6), 1023–1027. 10.1242/dev.102210 25758217 PMC4360182

[B5] Buckwalter J. A. Cooper R. R. (1987). Bone Structure and Function. Instr. Course Lect 36, 27–48. 3325555

[B6] Caplan A. I. (1991). Mesenchymal Stem Cells. J. Orthop. Res. 9 (5), 641–650. 10.1002/jor.1100090504 1870029

[B7] Caplan A. I. (2017). Mesenchymal Stem Cells: Time to Change the Name!. Stem Cell Transl Med 6 (6), 1445–1451. 10.1002/sctm.17-0051 PMC5689741 28452204

[B8] Chan C. K. F. Gulati G. S. Sinha R. Tompkins J. V. Lopez M. Carter A. C. (2018). Identification of the Human Skeletal Stem Cell. Cell 175 (1), 43–56. 10.1016/j.cell.2018.07.029 30241615 PMC6400492

[B9] Chan C. K. F. Seo E. Y. Chen J. Y. Lo D. McArdle A. Sinha R. (2015). Identification and Specification of the Mouse Skeletal Stem Cell. Cell 160 (1-2), 285–298. 10.1016/j.cell.2014.12.002 25594184 PMC4297645

[B10] Chanavaz M. (1995). The Periosteum: the "umbilical Cord" of Bone. Quantification of the Blood Supply of Cortical Bone of Periosteal Origin. Rev. Stomatol Chir Maxillofac. 96 (4), 262–267. 7569717

[B11] Choi I. H. Chung C. Y. Cho T. J. Yoo W. J. (2002). Angiogenesis and Mineralization during Distraction Osteogenesis. J. Korean Med. Sci. 17 (4), 435–447. 10.3346/jkms.2002.17.4.435 12172035 PMC3054899

[B12] Colnot C. (2009). Skeletal Cell Fate Decisions within Periosteum and Bone Marrow during Bone Regeneration. J. Bone Mineral Res. 24 (2), 274–282. 10.1359/jbmr.081003 PMC3276357 18847330

[B13] Crisan M. Yap S. Casteilla L. Chen C.-W. Corselli M. Park T. S. (2008). A Perivascular Origin for Mesenchymal Stem Cells in Multiple Human Organs. Cell Stem Cell 3 (3), 301–313. 10.1016/j.stem.2008.07.003 18786417

[B14] Debnath S. Yallowitz A. R. McCormick J. Lalani S. Zhang T. Xu R. (2018). Discovery of a Periosteal Stem Cell Mediating Intramembranous Bone Formation. Nature 562 (7725), 133–139. 10.1038/s41586-018-0554-8 30250253 PMC6193396

[B15] Diaz-Flores L. Gutierrez R. Lopez-Alonso A. Gonzalez R. Varela H. (1992). Pericytes as a Supplementary Source of Osteoblasts in Periosteal Osteogenesis. Clin. Orthopaedics Relat. Res. 275, 280–286. 10.1097/00003086-199202000-00042 1735226

[B16] Dominici M. Le Blanc K. Mueller I. Slaper-Cortenbach I. Marini F. C. Krause D. S. (2006). Minimal Criteria for Defining Multipotent Mesenchymal Stromal Cells. The International Society for Cellular Therapy Position Statement. Cytotherapy 8 (4), 315–317. 10.1080/14653240600855905 16923606

[B17] Duchamp de Lageneste O. Julien A. Abou-Khalil R. Frangi G. Carvalho C. Cagnard N. (2018). Periosteum Contains Skeletal Stem Cells with High Bone Regenerative Potential Controlled by Periostin. Nat. Commun. 9 (1), 773. 10.1038/s41467-018-03124-z 29472541 PMC5823889

[B18] Dwek J. R. (2010). The Periosteum: what Is it, where Is it, and what Mimics it in its Absence? Skeletal Radiol. 39 (4), 319–323. 10.1007/s00256-009-0849-9 20049593 PMC2826636

[B19] Esposito A. Wang L. Li T. Miranda M. Spagnoli A. (2020). Role of Prx1-Expressing Skeletal Cells and Prx1-Expression in Fracture Repair. Bone 139, 115521. 10.1016/j.bone.2020.115521 32629173 PMC7484205

[B20] Eyckmans J. Roberts S. J. Schrooten J. Luyten F. P. (2010). A Clinically Relevant Model of Osteoinduction: a Process Requiring Calcium Phosphate and BMP/Wnt Signalling. J. Cel Mol Med 14 (6B), 1845–1856. 10.1111/j.1582-4934.2009.00807.x PMC3829044 19538476

[B21] Ferguson C. Alpern E. Miclau T. Helms J. A. (1999). Does Adult Fracture Repair Recapitulate Embryonic Skeletal Formation? Mech. Dev. 87 (1-2), 57–66. 10.1016/s0925-4773(99)00142-2 10495271

[B22] Ferretti C. Mattioli-Belmonte M. (2014). Periosteum Derived Stem Cells for Regenerative Medicine Proposals: Boosting Current Knowledge. Wjsc 6 (3), 266–277. 10.4252/wjsc.v6.i3.266 25126377 PMC4131269

[B23] Gallardo-Calero I. Barrera-Ochoa S. Manzanares M. C. Sallent A. Vicente M. López-Fernández A. (2019). Vascularized Periosteal Flaps Accelerate Osteointegration and Revascularization of Allografts in Rats. Clin. Orthop. Relat. Res. 477 (4), 741–755. 10.1097/CORR.0000000000000400 30810538 PMC6437352

[B24] Gao B. Deng R. Chai Y. Chen H. Hu B. Wang X. (2019). Macrophage-lineage TRAP+ Cells Recruit Periosteum-Derived Cells for Periosteal Osteogenesis and Regeneration. J. Clin. Invest. 129 (6), 2578–2594. 10.1172/JCI98857 30946695 PMC6538344

[B25] Hanada K. Solchaga L. A. Caplan A. I. Hering T. M. Goldberg V. M. Yoo J. U. (2001). BMP-2 Induction and TGF-?1 Modulation of Rat Periosteal Cell Chondrogenesis. J. Cel. Biochem. 81 (2), 284–294. 10.1002/1097-4644(20010501)81:2<284:aid-jcb1043>3.0.co;2-d 11241668

[B26] Isern J. García-García A. Martín A. M. Arranz L. Martín-Pérez D. Torroja C. (2014). The Neural Crest Is a Source of Mesenchymal Stem Cells with Specialized Hematopoietic Stem Cell Niche Function. Elife 3, e03696. 10.7554/eLife.03696 25255216 PMC4381911

[B27] Jaloux C. Bettex Q. Levadoux M. Cerlier A. Iniesta A. Legre R. (2020). Free Vascularized Medial Femoral Condyle Corticoperiosteal Flap with Non-vascularized Iliac Crest Graft for the Treatment of Recalcitrant Clavicle Non-union. J. Plast. Reconstr. Aesthet. Surg. 73 (7), 1232–1238. 10.1016/j.bjps.2020.03.018 32414702

[B28] Jazrawi L. M. Majeska R. J. Klein M. L. Kagel E. Stromberg L. Einhorn T. A. (1998). Bone and Cartilage Formation in an Experimental Model of Distraction Osteogenesis. J. Orthopaedic Trauma 12 (2), 111–116. 10.1097/00005131-199802000-00008 9503300

[B29] Julien A. Kanagalingam A. Martínez-Sarrà E. Megret J. Luka M. Ménager M. (2021). Direct Contribution of Skeletal Muscle Mesenchymal Progenitors to Bone Repair. Nat. Commun. 12 (1), 2860. 10.1038/s41467-021-22842-5 34001878 PMC8128920

[B30] Kobayashi S. Takebe T. Inui M. Iwai S. Kan H. Zheng Y.-W. (2011). Reconstruction of Human Elastic Cartilage by a CD44+ CD90+ Stem Cell in the Ear Perichondrium. Proc. Natl. Acad. Sci. 108 (35), 14479–14484. 10.1073/pnas.1109767108 21836053 PMC3167558

[B31] Kojimoto H. Yasui N. Goto T. Matsuda S. Shimomura Y. (1988). Bone Lengthening in Rabbits by Callus Distraction. The Role of Periosteum and Endosteum. The J. Bone Jt. Surg. Br. volume 70-B (4), 543–549. 10.1302/0301-620X.70B4.3403595 3403595

[B32] Kronenberg H. M. (2007). The Role of the Perichondrium in Fetal Bone Development. Ann. New York Acad. Sci. 1116, 59–64. 10.1196/annals.1402.059 18083921

[B33] Lapierre F. Masquelet A. Aesch B. Romana C. Goga D. (1991). Cranioplasties Using Free Femoral Osteo-Periostal Flaps. Chirurgie 117 (4), 293–297. 1817825

[B34] Liu R. Birke O. Morse A. Peacock L. Mikulec K. Little D. G. (2011). Myogenic Progenitors Contribute to Open but Not Closed Fracture Repair. BMC Musculoskelet. Disord. 12, 288. 10.1186/1471-2474-12-288 22192089 PMC3266223

[B35] Long F. (2011). Building strong Bones: Molecular Regulation of the Osteoblast Lineage. Nat. Rev. Mol. Cel Biol 13 (1), 27–38. 10.1038/nrm3254 22189423

[B36] Long F. Ornitz D. M. (2013). Development of the Endochondral Skeleton. Cold Spring Harbor Perspect. Biol. 5 (1), a008334. 10.1101/cshperspect.a008334 PMC3579395 23284041

[B37] Mach D. B. Rogers S. D. Sabino M. C. Luger N. M. Schwei M. J. Pomonis J. D. (2002). Origins of Skeletal Pain: Sensory and Sympathetic Innervation of the Mouse Femur. Neuroscience 113 (1), 155–166. 10.1016/s0306-4522(02)00165-3 12123694

[B38] Masquelet A. C. Romana M. C. Penteado C. V. Carlioz H. (1988). Vascularized Periosteal Grafts. Anatomic Description, Experimental Study, Preliminary Report of Clinical Experience]. Rev. Chir Orthop. Reparatrice Appar Mot. 74 Suppl 2 (Suppl. 2), 240–243. 3231789

[B39] Matsuo K. Ji S. Miya A. Yoda M. Hamada Y. Tanaka T. (2019). Innervation of the Tibial Epiphysis through the Intercondylar Foramen. Bone 120, 297–304. 10.1016/j.bone.2018.11.007 30439572

[B40] Matthews B. G. Grcevic D. Wang L. Hagiwara Y. Roguljic H. Joshi P. (2014). Analysis of αSMA-Labeled Progenitor Cell Commitment Identifies Notch Signaling as an Important Pathway in Fracture Healing. J. Bone Miner Res. 29 (5), 1283–1294. 10.1002/jbmr.2140 24190076 PMC4864015

[B41] Matthews B. G. Novak S. Sbrana F. V. Funnell J. L. Cao Y. Buckels E. J. (2021). Heterogeneity of Murine Periosteum Progenitors Involved in Fracture Healing. Elife 10. 10.7554/eLife.58534 PMC7906599 33560227

[B42] Méndez-Ferrer S. Michurina T. V. Ferraro F. Mazloom A. R. Macarthur B. D. Lira S. A. (2010). Mesenchymal and Haematopoietic Stem Cells Form a Unique Bone Marrow Niche. Nature 466 (7308), 829–834. 10.1038/nature09262 20703299 PMC3146551

[B43] Meyer M. B. Benkusky N. A. Sen B. Rubin J. Pike J. W. (2016). Epigenetic Plasticity Drives Adipogenic and Osteogenic Differentiation of Marrow-Derived Mesenchymal Stem Cells. J. Biol. Chem. 291 (34), 17829–17847. 10.1074/jbc.M116.736538 27402842 PMC5016174

[B44] Mizuhashi K. Ono W. Matsushita Y. Sakagami N. Takahashi A. Saunders T. L. (2018). Resting Zone of the Growth Plate Houses a Unique Class of Skeletal Stem Cells. Nature 563 (7730), 254–258. 10.1038/s41586-018-0662-5 30401834 PMC6251707

[B45] Moore E. R. Zhu Y. X. Ryu H. S. Jacobs C. R. (2018). Periosteal Progenitors Contribute to Load-Induced Bone Formation in Adult Mice and Require Primary Cilia to Sense Mechanical Stimulation. Stem Cel Res Ther 9 (1), 190. 10.1186/s13287-018-0930-1 PMC6042447 29996901

[B46] Nakahara H. Bruder S. P. Goldberg V. M. Caplan A. I. (1990). *In Vivo* osteochondrogenic Potential of Cultured Cells Derived from the Periosteum. Clin. Orthopaedics Relat. Res. 259, 223–232. 10.1097/00003086-199010000-00032 2208860

[B47] Nakashima K. Zhou X. Kunkel G. Zhang Z. Deng J. M. Behringer R. R. (2002). The Novel Zinc finger-containing Transcription Factor Osterix Is Required for Osteoblast Differentiation and Bone Formation. Cell 108 (1), 17–29. 10.1016/s0092-8674(01)00622-5 11792318

[B48] Ochareon P. Herring S. W. (2011). Cell Replication in Craniofacial Periosteum: Appositional vs. Resorptive Sites. J. Anat. 218 (3), 285–297. 10.1111/j.1469-7580.2010.01336.x 21223257 PMC3058215

[B49] Olsen B. R. Reginato A. M. Wang W. (2000). Bone Development. Annu. Rev. Cel Dev. Biol. 16, 191–220. 10.1146/annurev.cellbio.16.1.191 11031235

[B50] Ortinau L. C. Wang H. Lei K. Deveza L. Jeong Y. Hara Y. (2019). Identification of Functionally Distinct Mx1+αSMA+ Periosteal Skeletal Stem Cells. Cell Stem Cell 25 (6), 784–796. 10.1016/j.stem.2019.11.003 31809737 PMC7055207

[B51] Park D. Spencer J. A. Koh B. I. Kobayashi T. Fujisaki J. Clemens T. L. (2012). Endogenous Bone Marrow MSCs Are Dynamic, Fate-Restricted Participants in Bone Maintenance and Regeneration. Cell Stem Cell 10 (3), 259–272. 10.1016/j.stem.2012.02.003 22385654 PMC3652251

[B52] Peterson R. E. Hoffman S. Kern M. J. (2005). Opposing Roles of Two Isoforms of the Prx1 Homeobox Gene in Chondrogenesis. Dev. Dyn. 233 (3), 811–821. 10.1002/dvdy.20412 15895367

[B53] Pineault K. M. Song J. Y. Kozloff K. M. Lucas D. Wellik D. M. (2019). Hox11 Expressing Regional Skeletal Stem Cells Are Progenitors for Osteoblasts, Chondrocytes and Adipocytes throughout Life. Nat. Commun. 10 (1), 3168. 10.1038/s41467-019-11100-4 31320650 PMC6639390

[B54] Rana R. S. Wu J. S. Eisenberg R. L. (2009). Periosteal Reaction. Am. J. Roentgenology 193 (4), W259–W272. 10.2214/AJR.09.3300 19770293

[B55] Ransom R. C. Carter A. C. Salhotra A. Leavitt T. Marecic O. Murphy M. P. (2018). Mechanoresponsive Stem Cells Acquire Neural Crest Fate in Jaw Regeneration. Nature 563 (7732), 514–521. 10.1038/s41586-018-0650-9 30356216 PMC6481292

[B56] Rauch A. Haakonsson A. K. Madsen J. G. S. Larsen M. Forss I. Madsen M. R. (2019). Osteogenesis Depends on Commissioning of a Network of Stem Cell Transcription Factors that Act as Repressors of Adipogenesis. Nat. Genet. 51 (4), 716–727. 10.1038/s41588-019-0359-1 30833796

[B57] Runyan C. M. Gabrick K. S. (2017). Biology of Bone Formation, Fracture Healing, and Distraction Osteogenesis. J. Craniofac. Surg. 28 (5), 1380–1389. 10.1097/SCS.0000000000003625 28562424

[B58] Sasaki A. Mizuno M. Ozeki N. Katano H. Otabe K. Tsuji K. (2018). Canine Mesenchymal Stem Cells from Synovium Have a Higher Chondrogenic Potential Than Those from Infrapatellar Fat Pad, Adipose Tissue, and Bone Marrow. PLoS One 13 (8), e0202922. 10.1371/journal.pone.0202922 30138399 PMC6107231

[B59] Squier C. A. Ghoneim S. Kremenak C. R. (1990). Ultrastructure of the Periosteum from Membrane Bone. J. Anat. 171, 233–239. 2081707 PMC1257144

[B60] Steinbrech D. S. Mehrara B. J. Rowe N. M. Dudziak M. E. Luchs J. S. Saadeh P. B. (2000). Gene Expression of TGF-??, TGF-?? Receptor, and Extracellular Matrix Proteins during Membranous Bone Healing in Rats. Plast. Reconstr. Surg. 105 (6), 2028–2038. 10.1097/00006534-200005000-00018 10839400

[B61] Stich S. Loch A. Leinhase I. Neumann K. Kaps C. Sittinger M. (2008). Human Periosteum-Derived Progenitor Cells Express Distinct Chemokine Receptors and Migrate upon Stimulation with CCL2, CCL25, CXCL8, CXCL12, and CXCL13. Eur. J. Cel Biol. 87 (6), 365–376. 10.1016/j.ejcb.2008.03.009 18501472

[B62] ten Berge D. Brouwer A. Korving J. Martin J. F. Meijlink F. (1998). Prx1 and Prx2 in Skeletogenesis: Roles in the Craniofacial Region, Inner Ear and Limbs. Development 125 (19), 3831–3842. 10.1242/dev.125.19.3831 9729491

[B63] Tikhonova A. N. Dolgalev I. Hu H. Sivaraj K. K. Hoxha E. Cuesta-Domínguez Á. (2019). The Bone Marrow Microenvironment at Single-Cell Resolution. Nature 569 (7755), 222–228. 10.1038/s41586-019-1104-8 30971824 PMC6607432

[B64] Tournaire G. Stegen S. Giacomini G. Stockmans I. Moermans K. Carmeliet G. (2020). Nestin-GFP Transgene Labels Skeletal Progenitors in the Periosteum. Bone 133, 115259. 10.1016/j.bone.2020.115259 32036051

[B65] Tsuji K. Bandyopadhyay A. Harfe B. D. Cox K. Kakar S. Gerstenfeld L. (2006). BMP2 Activity, Although Dispensable for Bone Formation, Is Required for the Initiation of Fracture Healing. Nat. Genet. 38 (12), 1424–1429. 10.1038/ng1916 17099713

[B66] Uddströmer L. (1978). The Osteogenic Capacity of Tubular and Membranous Bone Periosteum: A Qualitative and Quantitative Experimental Study in Growing Rabbits. Scand. J. Plast. Reconstr. Surg. 12 (3), 195–205. 10.3109/02844317809012995 368969

[B67] Ueno T. Kagawa T. Mizukawa N. Nakamura H. Sugahara T. Yamamoto T. (2001). Cellular Origin of Endochondral Ossification from Grafted Periosteum. Anat. Rec. 264 (4), 348–357. 10.1002/ar.10024 11745090

[B68] Viswanathan S. Shi Y. Galipeau J. Krampera M. Leblanc K. Martin I. (2019). Mesenchymal Stem versus Stromal Cells: International Society for Cell & Gene Therapy (ISCT) Mesenchymal Stromal Cell Committee Position Statement on Nomenclature. Cytotherapy 21 (10), 1019–1024. 10.1016/j.jcyt.2019.08.002 31526643

[B69] Wang Y. Chen L. Kang M. Ling L. Tian F. Won‐Kim S. H. (2019). The Fracture Callus Is Formed by Progenitors of Different Skeletal Origins in a Site‐Specific Manner. JBMR Plus 3 (9), e10193. 10.1002/jbm4.10193 31667451 PMC6808225

[B70] Wong S.-P. Rowley J. E. Redpath A. N. Tilman J. D. Fellous T. G. Johnson J. R. (2015). Pericytes, Mesenchymal Stem Cells and Their Contributions to Tissue Repair. Pharmacol. Ther. 151, 107–120. 10.1016/j.pharmthera.2015.03.006 25827580

[B71] Worthley D. L. Churchill M. Compton J. T. Tailor Y. Rao M. Si Y. (2015). Gremlin 1 Identifies a Skeletal Stem Cell with Bone, Cartilage, and Reticular Stromal Potential. Cell 160 (1-2), 269–284. 10.1016/j.cell.2014.11.042 25594183 PMC4436082

[B72] Yachouh J. Breton P. Roux J.-P. Goudot P. (2010). Osteogenic Capacity of Vascularised Periosteum: an Experimental Study on Mandibular Irradiated Bone in Rabbits. J. Plast. Reconstr. Aesthet. Surg. 63 (12), 2160–2167. 10.1016/j.bjps.2010.01.015 20194051

[B73] Yiannakopoulos C. K. Kanellopoulos A. D. Trovas G. P. Dontas I. A. Lyritis G. P. (2007). The Biomechanical Capacity of the Periosteum in Intact Long Bones. Arch. Orthop. Trauma Surg. 128 (1), 117–120. 10.1007/s00402-007-0433-5 17874324

[B74] Zhang X. Xie C. Lin A. S. Ito H. Awad H. Lieberman J. R. (2005). Periosteal Progenitor Cell Fate in Segmental Cortical Bone Graft Transplantations: Implications for Functional Tissue Engineering. J. Bone Miner Res. 20 (12), 2124–2137. 10.1359/JBMR.050806 16294266 PMC4527562

[B75] Zhou B. O. Yue R. Murphy M. M. Peyer J. G. Morrison S. J. (2014). Leptin-receptor-expressing Mesenchymal Stromal Cells Represent the Main Source of Bone Formed by Adult Bone Marrow. Cell Stem Cell 15 (2), 154–168. 10.1016/j.stem.2014.06.008 24953181 PMC4127103

